# Injectable hybrid nanofibrous spheres made of PLA and nano-hydroxyapatite for cell delivery and osteogenic induction

**DOI:** 10.3389/fbioe.2024.1460870

**Published:** 2024-08-29

**Authors:** Yawen Wang, Xiaopei Zhang, Na Liu, Renjie Chen, Chenghao Yu, Lijie Yao, Siyu Chen, Yuying Yan, Tong Wu, Yuanfei Wang

**Affiliations:** ^1^ Shandong Key Laboratory of Medical and Health Textile Materials, Collaborative Innovation Center for Eco-textiles of Shandong Province and the Ministry of Education, College of Textile and Clothing, Qingdao University, Qingdao, China; ^2^ Medical Research Center, The Affiliated Hospital of Qingdao University, Qingdao University, Qingdao, China; ^3^ Institute of Neuroregeneration and Neurorehabilitation, Department of Pathophysiology, School of Basic Medicine, Qingdao University, Qingdao, China; ^4^ Beijing Jishuitan Hospital, Capital Medical University, Beijing, China; ^5^ Qingdao Stomatological Hospital Affiliated to Qingdao University, Qingdao, China

**Keywords:** nanofibrous spheres, osteogenic, nano-hydroxyapatite, cell delivery, injectable

## Abstract

**Introduction:**

Nanofibrous spheres, with their injectable format and biomimetic three-dimensional topologies that emulate the complexity of natural extracellular environments, have become increasingly attractive for applications in biomedical and regenerative medicine. Our research contributes to this growing field by detailing the design and fabrication of a novel series of polylactic acid/nano-hydroxyapatite (PLA/nHA) hybrid nanofibrous spheres.

**Methods:**

These advanced structures were created by integrating electrospinning and electrospray techniques, which allowed for precise control over the nanofibrous spheres, especially in size. We have conducted a comprehensive investigation into the nanofibrous spheres’ capacity to deliver stem cells efficiently and maintain their viability post-implantation, as well as their potential to induce osteogenic differentiation.

**Results and Discussion:**

The results show that these nanofibrous spheres are biocompatible and injectable, effectively supporting the attachment, growth, and differentiation of bone marrow-derived mesenchymal stem cells while aiding in their targeted transportation to bone defect areas to execute their regenerative functions. The findings of this study could significantly impact the future development of biocompatible materials for a range of therapeutic applications, including bone tissue engineering and regenerative therapy.

## 1 Introduction

Minimally invasive therapies, in contrast to traditional surgical methods, offer distinct advantages such as expedited recovery, reduced complications, and smaller incisions, making them increasingly regarded as promising strategies for addressing bone defects (Zhou et al., 2019; Ashammakhi et al.). An optimal scaffold for bone tissue engineering should exhibit desirable biocompatibility and a structure that emulates the natural skeletal architecture at the nanoscale or macroscale while providing a microenvironment that supports cell migration, proliferation, and differentiation (Zhang et al., 2019; Yu et al., 2022). Among others, injectable biomaterials represent a novel stage in minimally invasive therapy, offering the capability to deliver biologics and drugs, integrate cells at precise injury sites, and facilitate the controlled release of payloads (Castillo-Orozco et al., 2017). Unlike implantable scaffolds, injectable spheres are capable of filling and repairing bone defects with irregular shapes by simply adjusting parameters related to their preparation and functionalization, thereby creating a local microenvironment at the defect site that fosters tissue regeneration, accelerates the repair process, and reduces the risk of infection (Raucci et al., 2020; Fang et al., 2014). As previously mentioned, nanofibrous spheres are considered exemplary cell or therapeutic carriers due to their injectability and biomimetic microstructures (Zhou et al., 2016; Zhang et al., 2015). It is widely acknowledged that nanofibers possess a large specific surface area and a structure reminiscent of the extracellular matrix (ECM), making injectable spheres with nanofibrous structures highly suitable for use as carriers to enhance cell viability and tissue regeneration (Liu et al., 2023a). Regarding the materials used to fabricate nanofibrous spheres, polylactic acid (PLA), a biodegradable material approved by the FDA, has been manufactured into various bone tissue engineering scaffolds (Liu et al., 2011; Zhang et al., 2016; Yu et al., 2024). Despite the excellent tissue regeneration potential of pure PLA or PLA-based scaffolds, their application in osteogenesis has been limited due to insufficient regenerative potential.

**FIGURE 1:**
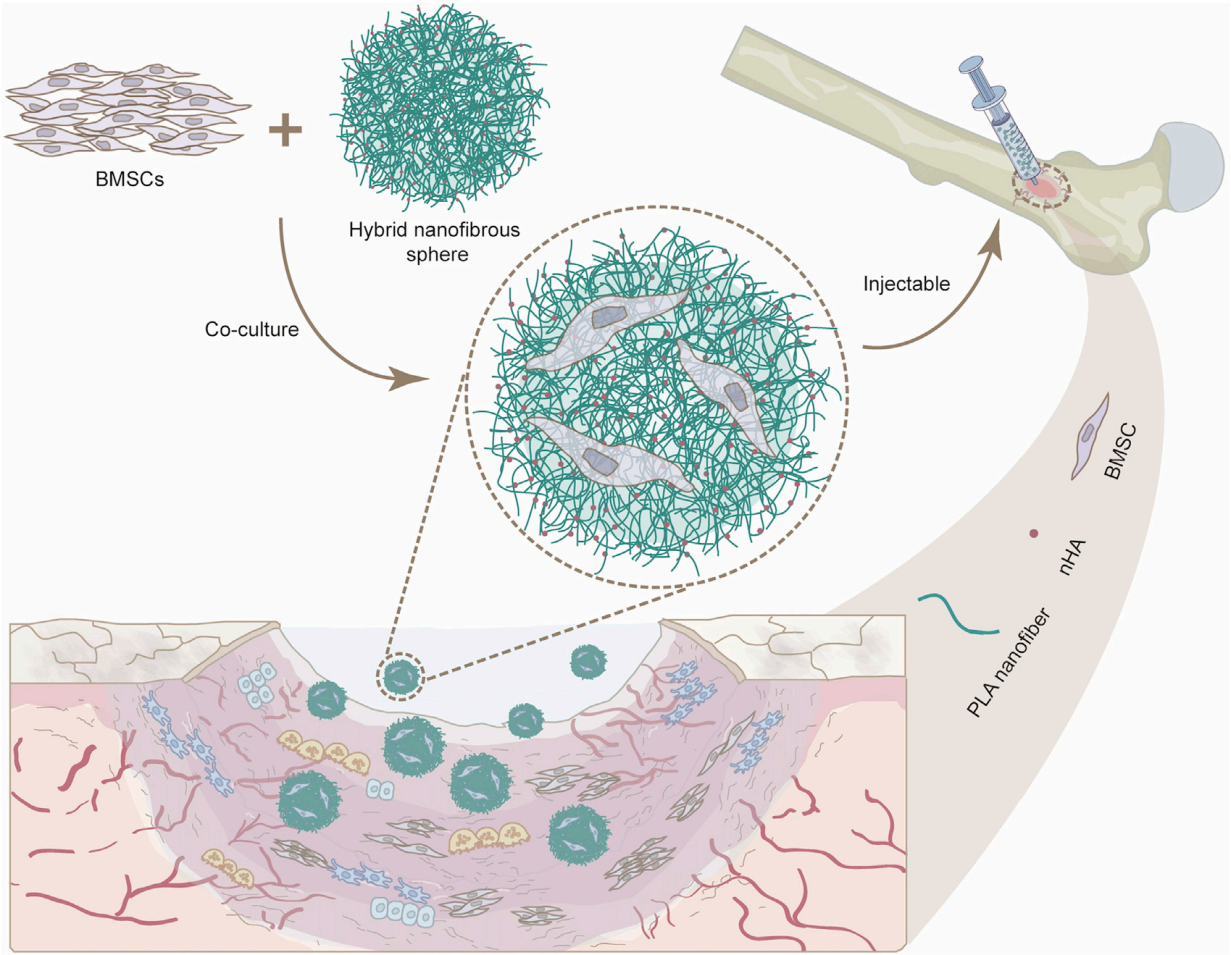
Schematic illustration showing the potential use of injectable nanofibrous spheres for bone defect repair.

To replicate the intricate bionic structure of natural bone and enhance the osteogenic potential while maintaining the flexibility of minimally invasive therapies, we first developed a novel class of injectable hybrid nanofibrous spheres by electrospinning and electrospray techniques, which synergistically combines nano-hydroxyapatite (nHA), a principal inorganic constituent of natural bone (Deng et al., 2024), with PLA for improving its biocompatibility (Liu et al., 2023b). The integration of nHA enhanced the scaffold’s osteoinductive potential and created a roughened surface. In addition, nHA displays basic properties that can neutralize the acidic byproducts generated during PLA degradation, thereby modulating the cellular survival state. The resultant nanofibrous spheres featured a rough, porous structure facilitating the transport of nutrients essential for cellular growth. Stem cells, especially bone marrow mesenchymal stem cells (BMSCs), can readily adhere to these nanofibrous spheres and proliferate. As such, the nanofibrous spheres’ injectability will allow for the precise delivery of BMSCs to bone defects, thereby catalyzing bone regeneration (Figure 1). Our study meticulously assessed the adhesion and proliferation of MC3T3 cells and BMSCs on these nanofibrous spheres, as well as their osteogenic potential when nHA is integrated, offering fresh perspectives for the repair of *in-situ* bone defects.

## 2 Materials and methods

### 2.1 Materials and reagents

PLA (Mw ≈ 150,000 g mol^−1^) was obtained from the Shandong Academy of Pharmaceutical Science. nHA was purchased from Match Biomaterials Co., Ltd. (Shenzhen, China). Hexafluoro-2-propanol (HFIP) was bought from Shanghai Macklin Biochemical Company. Gelatin was obtained from Sigma-Aldrich (USA). Cell counting kit-8 (CCK-8) was purchased from GlpBio (USA). Alkaline phosphatase (ALP) assay kit was obtained from Applygen (Beijing, China). Phalloidin-iFluor 488 was supplied by Abcam (Shanghai, China). 4′,6-diamidino-2-phenylindole (DAPI) were acquired from Solarbio (Beijing, China). Fetal bovine serum (FBS) was obtained from Pan (Germany). Dulbecco’s modified eagle medium (DMEM) and antibiotic-antimycotic were purchased from Solarbio (Beijing, China). Osteogenic differentiation complete medium was purchased from HyCyTe (China). MC3T3 cells and BMSCs were provided by the “Advanced Biomaterials and Regenerative Medicine” Innovation Team.

### 2.2 Preparation of uniaxially aligned PLA/nHA hybrid nanofibers

nHA was ultrasonically dispersed in HFIP with a concentration of 5%, 10%, and 15% (w/v) for 90 min, in which PLA was dissolved with a concentration of 20% (w/v). After the mixture was completely dissolved, the obtained solution with different concentrations of nHA and 20% PLA was ultrasonically dispersed for another 90 min to be prepared for electrospinning. PLA/nHA hybrid nanofibers were produced by ET-2535H electrospinning equipment with an 11 kV voltage and a flow rate of 1 mL/h. A high-speed collection roller at 2,800 rpm was used to collect hybrid fibers, and the distance between the injection syringe and the roller was 15 cm. The pure PLA nanofiber (0% nHA) was prepared precisely with the same method.

### 2.3 Preparation of injectable PLA/nHA hybrid nanofibrous spheres

Different PLA/nHA hybrid nanofibers (0%, 5%, 10%, and 15% nHA) were cut to uniform-sized short hybrid nanofibers with Leica CM3050 Cryostat, followed by washing with deionized water and centrifugation at 2,000 rpm for 5 min. Then, the short fibers were mixed with prepared gelatin aqueous solution to obtain a suspension of 20 mg/mL short fibers, and the mass of gelatin was 5% of the short fibers. Afterward, these suspensions were directly electrosprayed into liquid nitrogen at different voltages to make nanofibrous spheres with different sizes based on PLA/nHA hybrid fiber. Under conditions where all other parameters remain equal, an increase in the applied electrospinning voltage results in a proportional reduction in the diameter of the nanofibrous spheres. Finally, these nanofibrous spheres were freeze-dried and crosslinked by glutaraldehyde to obtain a stable structure.

### 2.4 Characterization of PLA/nHA hybrid nanofibers and PLA/nHA hybrid nanofibrous spheres

To clarify the orientation of hybrid nanofibers and the morphologies of hybrid nanofibrous spheres, we plated Au/Pd on each sample with an ion sputtering instrument (BV10044, KYKY Technology Co., Ltd.), after that, hybrid fibers and nanofibrous spheres was observed, imaged and analyzed through a scanning electron microscope (SEM, PW-100-515, ThermoFisher). The average diameters of fibers were examined and recorded using ImageJ software.

### 2.5 Cell viability and morphology on uniaxially aligned PLA/nHA hybrid nanofibers

MC3T3 cells and BMSCs were inoculated in 24-well plates at a density of 5 × 10^3^ cells/well on different groups of hybrid fibers (0%, 5%, 10%, and 15% nHA) and TCP. After being cultured with different PLA/nHA hybrid fibers and TCP for 1, 3, and 5 days in a DMEM containing 10% FBS, the viability of MC3T3 cells and BMSCs was detected using the CCK-8 kit by measuring the absorbance of different samples at 450 nm through a spectrophotometric microplate reader. Afterward, cells on days 3 and 5 were fixed with 4% paraformaldehyde and stained by immunofluorescence to observe the effect of PLA/nHA hybrid fibers on their morphology. At the same time, the morphology of MC3T3 cells and BMSCs was also observed by SEM after dehydration through graded ethanol.

### 2.6 BMSCs viability on injectable PLA/nHA hybrid nanofibrous spheres

The experiment methods were performed in the same way as described above for PLA/nHA hybrid fibers, replacing the hybrid fibers with different groups of PLA/nHA nanofibrous spheres (0%, 5%, 10%, and 15% nHA) in uniform size, 10 nanofibrous spheres/well. On days 1, 3, and 5, the viability of BMSCs was detected by CCK-8 assay.

### 2.7 *In vitro* osteogenesis of the injectable PLA/nHA hybrid nanofibrous spheres

ALP assay kit was used to evaluate the *in vitro* osteogenic activity of different PLA/nHA nanofibrous spheres (0%, 5%, 10%, and 15% nHA). Nanofibrous spheres in uniform size were plated in a 24-well plate at a density of 10 spheres/well, and then BMSCs were inoculated at a density of 2 × 10^4^ cells/well on these nanofibrous spheres in DMEM containing 10% FBS. After 3 days of culture, the DMEM was replaced by Osteogenic differentiation complete medium. After 7 and 14 days of osteogenic differentiation induction, ALP activity on different groups of nanofibrous spheres was detected by measuring the absorbance of different samples at 405 nm through a spectrophotometric microplate reader.

### 2.8 Cell morphology on injectable PLA/nHA hybrid nanofibrous spheres

In order to investigate the effect of injectable PLA/nHA nanofibrous spheres on stem cell delivery and the influence on cell morphology, MC3T3 cells, and BMSCs were seeded on these nanofibrous spheres at a density of 1 × 10^4^ cells/well in a 96-well plate. After 3 and 5 days of incubation, MC3T3 cells and BMSCs were fixed with 4% paraformaldehyde, stained by immunofluorescence, and observed by a laser scanning confocal microscope (Nikon C2+, Japan), respectively.

### 2.9 Statistical analysis

The multiple comparison procedures between groups were performed using one-way ANOVA with Origin 2018, and each group was repeated at least three times. Statistical results were expressed as means ± standard deviation (SD). To observe the significance of differences between the test groups. Student's t-test was used for all two-by-two comparisons. A value of *p < 0.05 represents the lowest significance level, **p < 0.01 represents a moderate significance level, and ***p < 0.001 represents the highest significance level.

## 3 Results and discussion

### 3.1 Characterization of PLA/nHA hybrid nanofibers

**FIGURE 2:**
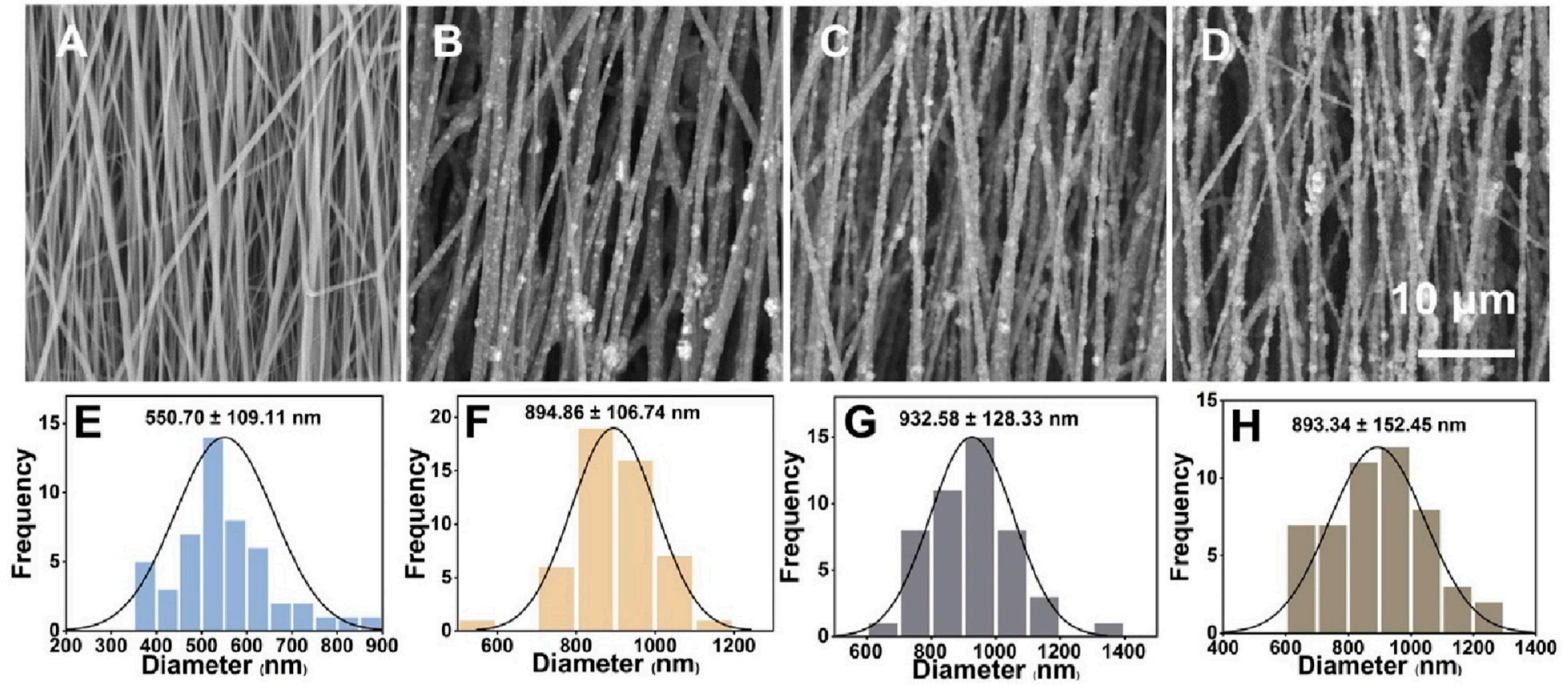
**(A–D)** SEM images of the uniaxially aligned PLA/nHA hybrid nanofibers containing 0%, 5%, 10%, and 15% nHA, respectively. **(E–H)** Diameter distribution of the uniaxially aligned PLA/nHA hybrid nanofibers.

Capitalizing on both PLA and nHA properties, we fabricated PLA/nHA hybrid nanofibers by electrospinning. Representative SEM images of nanofibers with different nHA content (0%, 5%, 10%, and 15%) and their normal distribution of diameters are given in Figure 2. It can be seen that nHA was successfully distributed in the nanofibers, and the fiber diameter was 550.70 ± 109.11 nm, 894.86 ± 106.74 nm, 932.58 ± 128.33 nm, and 893.34 ± 152.45 nm, respectively, from 0% to 15%. The presence of nHA between 5% and 15% in nanofibers did not markedly alter the diameters. Nonetheless, these fibers exhibited a significant increase in diameter compared to PLA nanofibers due to nHA integration. SEM images revealed a rougher surface on the nHA-containing hybrid fibers. These rough surfaces were expected to serve as topographic cues to provide contact guidance for cells and directly affect cell behavior at the cellular and subcellular levels (Jiao et al., 2022; Xue et al., 2019). Additionally, optimal fiber morphologies were achieved in the 5% nHA group. Higher nHA concentrations of 10% and 15% led to difficulties in achieving uniform dispersion, resulting in fiber aggregation, which compromised the fibers’ structural and surface properties.

### 3.2 Characterization of PLA/nHA hybrid nanofibrous spheres

**FIGURE 3:**
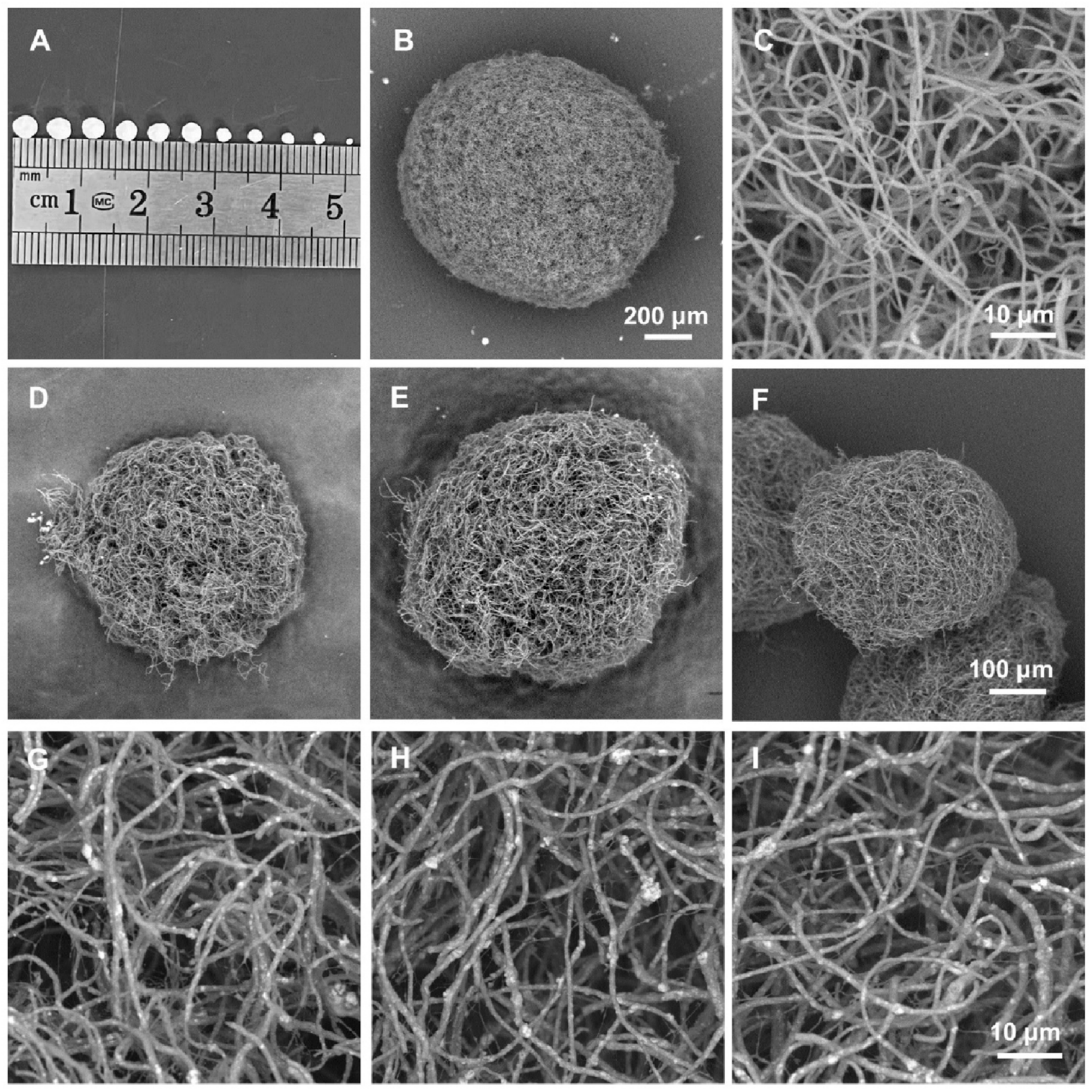
**(A)** Digital photographs of the hybrid nanofibrous spheres with different diameters. **(B, C)** SEM images of a pure PLA nanofibrous spheres (0%) and partially enlarged view of the short nanofibers in it. **(D–F)** SEM images of PLA/nHA hybrid nanofibrous spheres prepared with different PLA/nHA nanofibers (5%, 10%, and 15%, respectively). **(G–I)** SEM images of the enlarged view of PLA/nHA nanofibers in the different nanofibrous spheres (5%, 10%, and 15%, respectively).

Utilizing the hybrid nanofibers, we designed and fabricated a series of injectable and size-adjustable PLA/nHA hybrid nanofibrous spheres. Digital photographs and SEM images show the adjustable sizes (Figure 3A) and specific microstructures (Figures 3B–I). The diameter of these nanofibrous spheres can be adjusted by altering the electrospray parameters to fit various defect sizes, with the diameter in the range from 340 to 3,500 µm. Figure 3B exhibits the nanofibrous spheres prepared from pure PLA nanofibers (0%) featuring a porous surface structure, and the PLA nanofibers consisting of the nanofibrous spheres were shown in the magnified SEM image (Figure 3C). The individual nanofibers within the nanofibrous spheres preserved the ECM-mimicking characteristics. Figures 3D–F show the nanofibrous spheres fabricated from hybrid nanofibers with different nHA proportions (5%, 10%, and 15%), which, consistent with the 0% group, displayed a preserved porous structure on the surfaces. Research has suggested that porous structures of nanofibrous spheres can facilitate the accommodation of cells and the delivery of oxygen and nutrients, thereby enhancing cell proliferation (Kankala et al., 2019). High-magnification SEM images in Figures 3G–I detail an even distribution of nHA within the short nanofibers, creating a rough surface and a rugged secondary structure that will enhance cell attachment and proliferation (Liu et al., 2023c). These images confirmed that the nanofibrous spheres maintain their porous structure and macroscopic morphology regardless of the nHA concentration. Notably, the microscopic morphology was also maintained across the nanofibrous spheres of different sizes but with the same nHA concentration (Supplementary Figure S1). For the proof-of-concept demonstration, nanofibrous spheres with a diameter of 1467 ± 127 µm were selected for subsequent *in vitro* cell experiments.

### 3.3 Cell viability and morphology on PLA/nHA hybrid nanofibers

**FIGURE 4:**
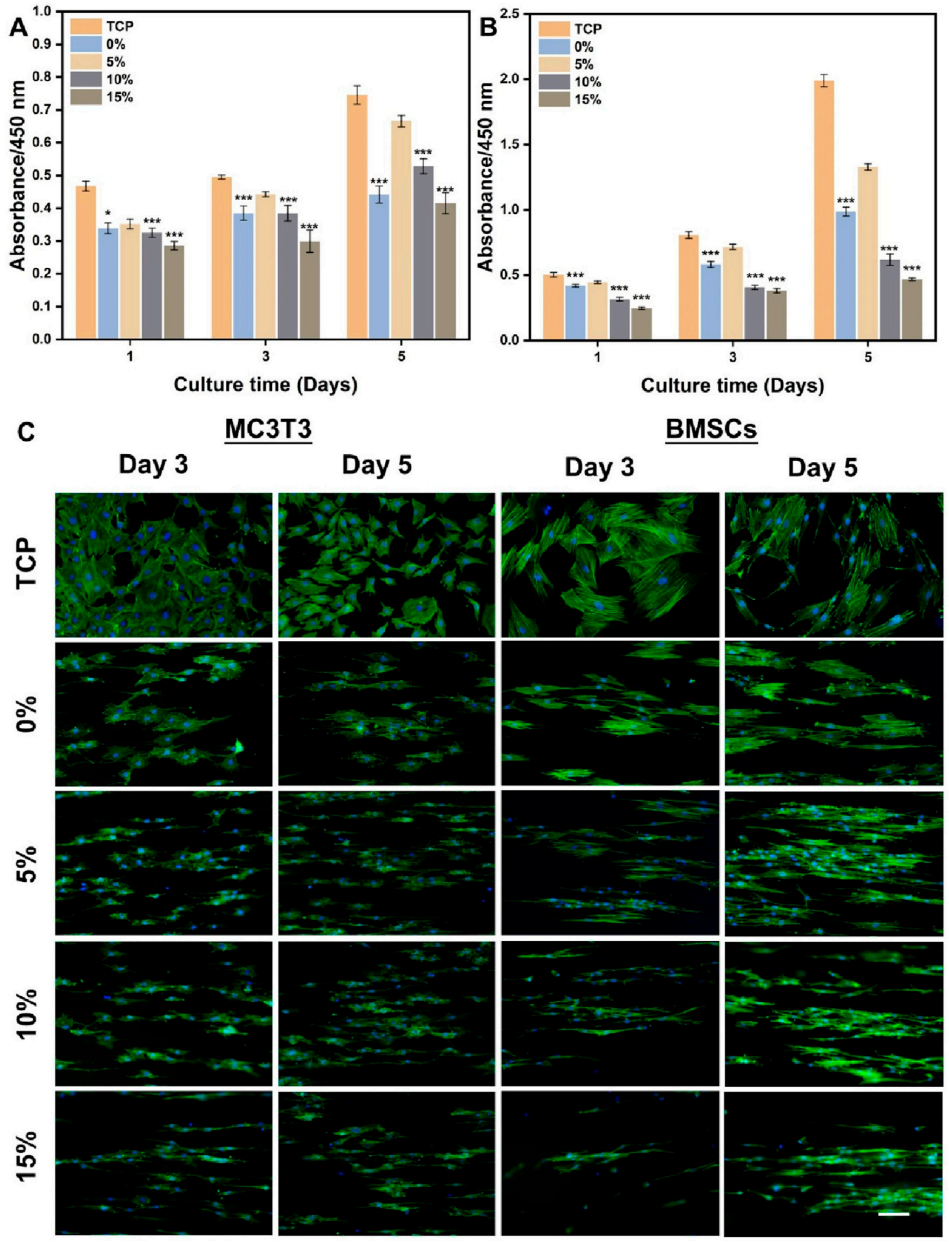
**(A)** Cell viability of MC3T3 cells cultured on the different PLA/nHA hybrid nanofibers and TCP on days 1, 3, and 5. **(B)** Cell viability of BMSCs cultured on the different PLA/nHA hybrid nanofibers and TCP on days 1, 3, and 5. *p < 0.05, **p < 0.01, ***p < 0.001 as compared with group 5%. **(C)** Fluorescence micrographs of MC3T3 cells and BMSCs cultured on the different PLA/nHA hybrid nanofibers on days 3 and 5. Blue: DAPI for staining cell nucleus; Green: Phalloidin-iFluor 488 for staining cytoskeleton. Scale bar = 100 μm, and it applies to all images.

**FIGURE 5:**
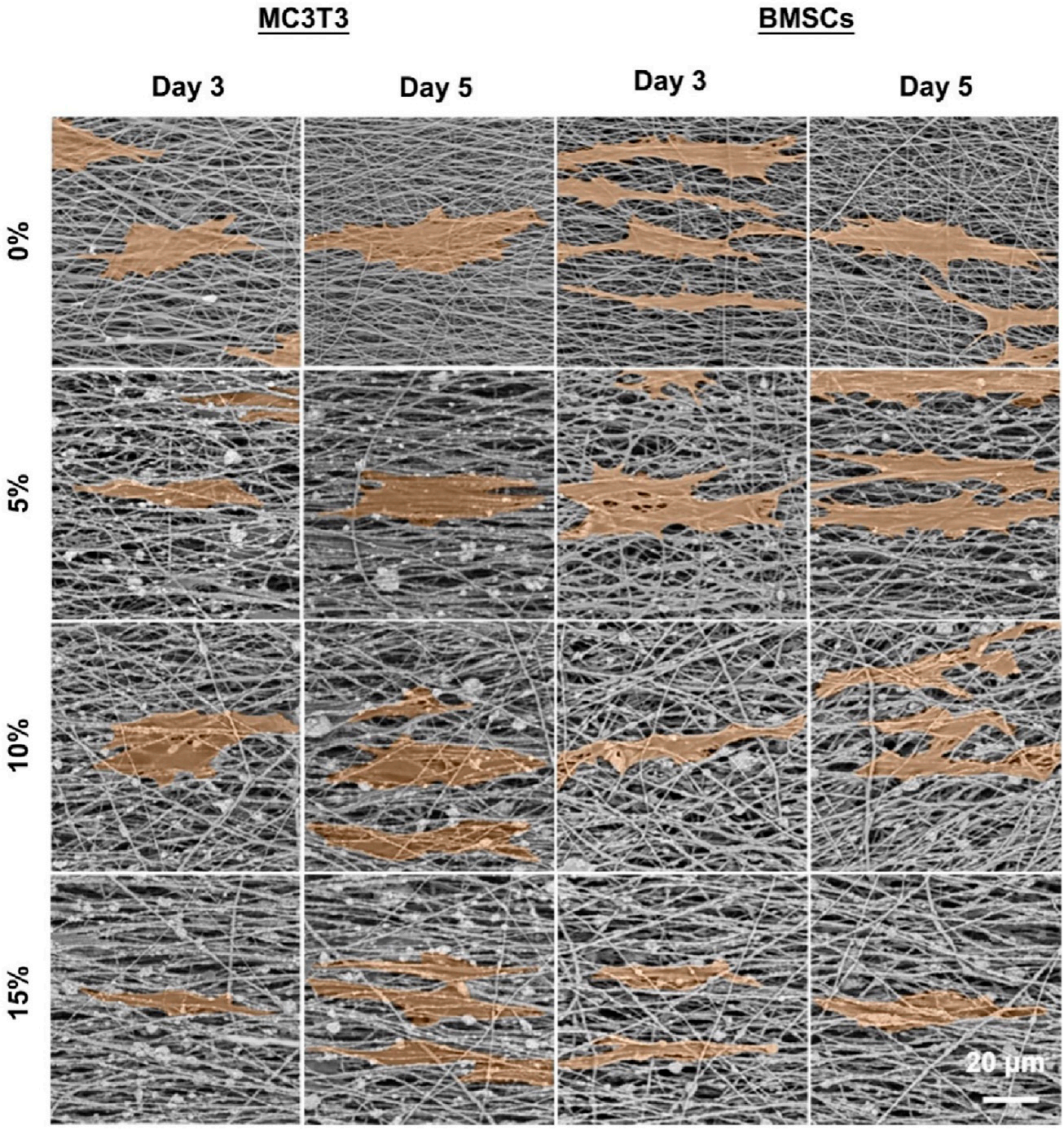
SEM images of MC3T3 cells and BMSCs cultured on the different PLA/nHA nanofibers on days 3 and 5. The cells were highlighted in orange for easy observation.

The effects of hybrid nanofibers on cell behavior were first explored by seeding MC3T3 cells and BMSCs into different groups of nanofibers. Cell viability and morphology were analyzed over time, with cell viability assays performed on days 1, 3, and 5 and morphological assessments conducted on days 3 and 5. The proliferation of MC3T3 cells and BMSCs on different nanofibers was demonstrated in Figures 4A,B. We observed that the cells kept proliferation on days 1, 3, and 5, suggesting favorable biocompatibility of PLA/nHA hybrid nanofibers. Specifically, the 5% nHA group exhibited the fastest proliferation rate among all hybrid nanofibers. Figure 4C display the morphology of MC3T3 cells and BMSCs cultured on the different nanofibers on days 3 and 5. Cells on the hybrid nanofibers exhibited extended and aligned fashions, with their cytoskeletal proteins aligned parallel to the nanofiber axis. Including nHA in hybrid nanofibers imparted a “slenderer” cell appearance, likely due to the enhanced roughness from nHA, which is beneficial to cell adhesion and extension (Xue et al., 2019; Li et al., 2010). Moreover, the SEM images of cells on fibers (Figure 5) indicates that the hybrid nanofibers with an appropriate nHA content are more effective in promoting cell adhesion. A high nHA content during the preparation process can lead to aggregation, which compromises the surface integrity of the nanofibers, potentially explaining the lower cytocompatibility observed in the 15% and 10% nHA groups compared to the 5% nHA group (Yu et al., 2022).

### 3.4 Cell viability, morphology, and osteogenic induction on PLA/nHA hybrid nanofibrous spheres

**FIGURE 6:**
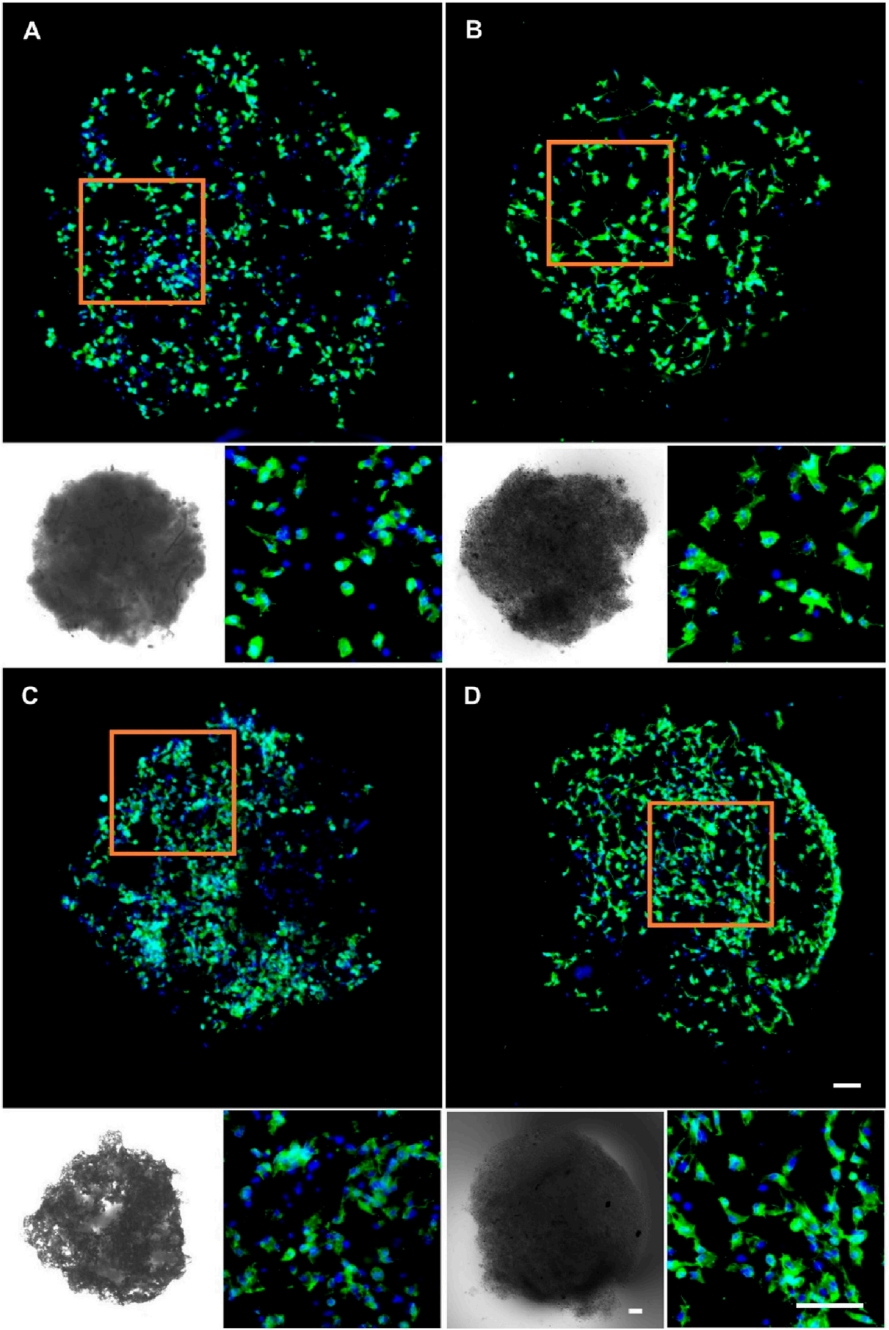
Fluorescence micrographs of MC3T3 cells cultured on **(A)** 0% nHA nanofibrous spheres of day 3, **(B)** 5% nHA nanofibrous spheres of day 3, **(C)** 0% nHA nanofibrous spheres of day 5, and **(D)** 5% nHA nanofibrous spheres of day 5. Blue: DAPI for staining cell nucleus; Green: Phalloidin-iFluor 488 for staining cytoskeleton; The lower left corner of each group is the light microscopy photo of the nanofibrous spheres. Scale bar = 100 μm, and it applies to all images.

Given the superior biocompatibility of the 5% nHA hybrid nanofibers, we investigated the effect of injectable nanofibrous spheres composed of these nanofibers on MC3T3 cell delivery and morphology, and pure PLA nanofibrous spheres were used as control. Cell distribution on the nanofibrous spheres was monitored on days 3 and 5 by laser confocal microscopy (Figure 6). A significant increase in the number and density of MC3T3 cells was observed on day 5, suggesting the robust adhesion and proliferation of cells on 5% nHA nanofibrous spheres group. 2D scaffold-based cell culture, followed by dissociation for injury treatment, lacks the complexity of the *in vivo* 3D microenvironment, leading to compromised site-specific targeting. Furthermore, direct injection of stem cells faced low survival rates and unintended migration, obstructing effective 3D tissue construction. Hence, the innovation of injectable spheres was critical (Wang et al., 2023). Nanofibrous spheres, in particular, may represent an optimal choice for the delivery of stem cells, as their 3D nanofibrous structures closely mimic the ECM niche of adult stem cells, thereby potentially enhancing stem cell homing efficacy and therapeutic outcomes (Boda et al., 2018). Besides, such nanofibrous spheres were designed with nHA-induced surface roughness, thus providing sufficient space for cell proliferation and facilitating nutrient transfer. The high specific surface area of the hybrid nanofibrous spheres supported cell proliferation effectively and offered promising potential as cell delivery carriers (Figures 1, 6).

**FIGURE 7:**
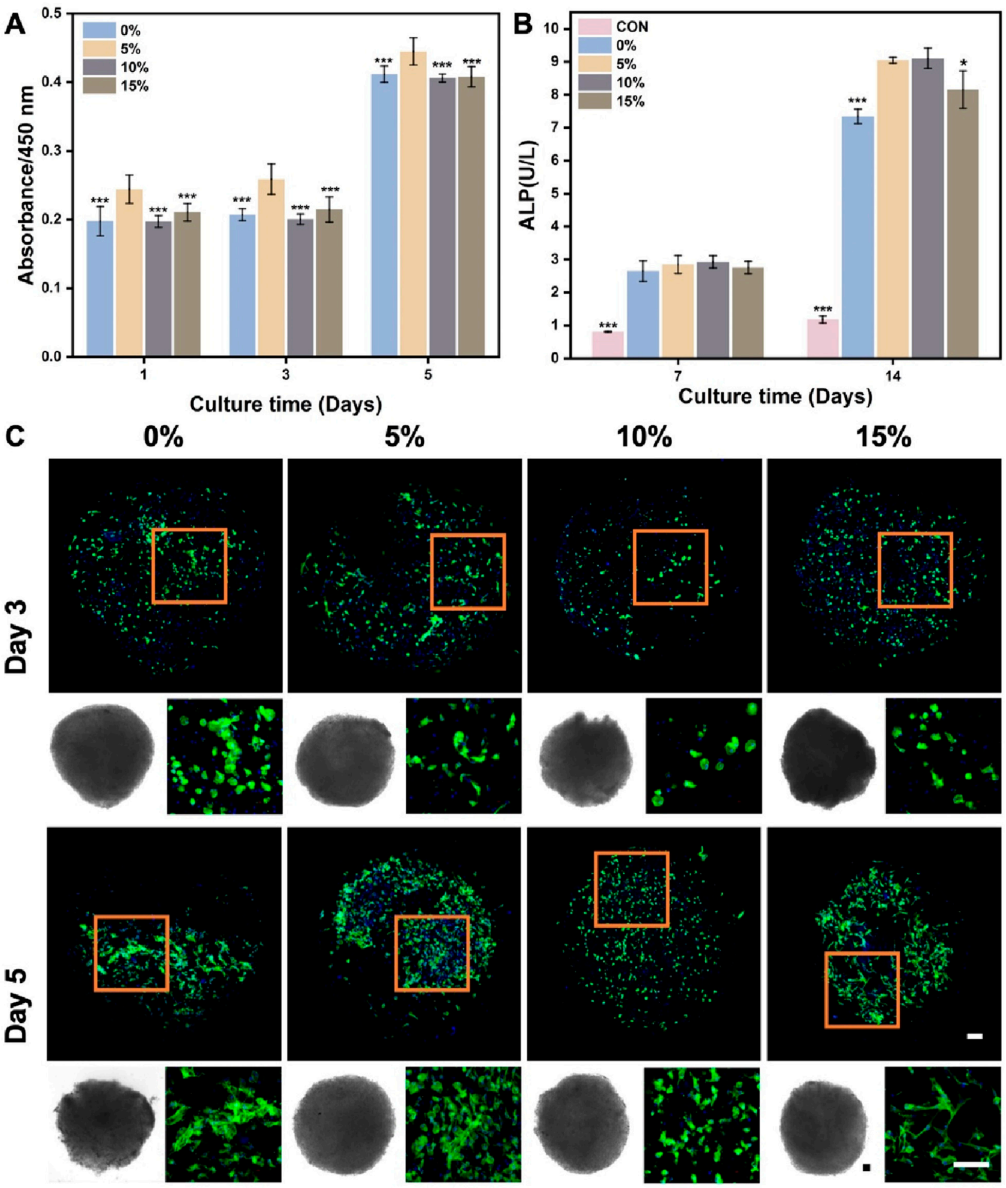
**(A)** Cell viability of BMSCs after cultured on the different PLA/nHA hybrid nanofibrous spheres on days 1, 3, and 5. *p < 0.05, **p < 0.01, ***p < 0.001 as compared with 5% group. **(B)** Quantitative analysis of ALP activity after 7 and 14 days osteogenic incubation of BMSCs on different PLA/nHA hybrid nanofibrous spheres. *p < 0.05, **p < 0.01, ***p < 0.001 as compared with group 5% and 10%. **(C)** Fluorescence micrographs of BMSCs after 3 and 5 days of culture on different PLA/nHA hybrid nanofibrous spheres. Blue: DAPI for staining cell nucleus; Green: Phalloidin-iFluor 488 for staining cytoskeleton; The lower left corner of each group is the light microscopy photo of the nanofibrous sphere. Scale bar = 100 μm, and it applies to all images.

In the context of using stem cell delivery for bone regeneration, our main goals were to evaluate BMSCs delivery efficiency and the influence of nanofibrous spheres on osteogenic differentiation. BMSCs were cultured on nanofibrous spheres with varying nHA levels, and viability was assessed on days 1, 3, and 5 (Figure 7A). Stable cell viability was observed, with a significant rise in cell count by day 5, suggesting effective BMSCs proliferation and the nanofibrous spheres’ favorable biocompatibility. The addition of nHA did not compromise biocompatibility, Remarkably, the 5% nHA group exhibited the highest cell viability, which was significantly different from the other nanofibrous spheres groups, conforming to the observed trends on the hybrid nanofibers.

ALP activity assessed the osteogenic differentiation of BMSCs co-cultured with nanofibrous spheres for 7 and 14 days. Figure 7B shows rising ALP levels from day 7 to 14, with nHA-containing nanofibrous spheres exhibiting higher levels than pure PLA ones after 14 days. This suggests that nHA enhances the osteogenic potential of nanofibrous spheres, with 5% and 10% nHA groups showing the most significant increases. Light and fluorescence microscopy (Figure 7C) revealed well-maintained nanofibrous spheres morphology and stable BMSCs adhesion, with observable cell growth and cytoskeletal development by day 5. These findings suggest that these hybrid nanofibrous spheres can support stem cell function, potentially enhancing stem cell delivery and stability.

Although hybrid nanofibrous spheres have been proven to be compatible cell carriers for various stem cell types, we still wish to emphasize their potential in bone regeneration. For the repair of large-area bone defects, there’s an urgent need to use injectable biomaterials combined with stem cells for minimally invasive treatment (Neves et al., 2016). Nanofibrous spheres, with their ECM-like features, show more significant potential for promoting osteogenic activity in bone regenerative cells than solid microspheres (Yan et al., 2022; Yang et al., 2022).

## 4 Conclusion

By combining PLA and nHA properties, we’ve developed a range of injectable PLA/nHA hybrid nanofibrous spheres, customizable in size and mimicking the natural ECM’s 3D topography, providing a textured surface for cell growth. These nanofibrous spheres exhibit excellent biocompatibility and injectability, supporting BMSCs adhesion, proliferation and differentiation, aiding their transport to and function at bone defect sites. The 5% nHA nanofibrous spheres stood out for their biocompatibility and ALP activity. Future refinements will involve adding biological cues like growth factors or drugs to enhance their performance in stem cell delivery and osteogenic differentiation. This approach presents a promising strategy for treating bone defects and can be adapted for various stem cell deliveries, potentially benefiting the regeneration of nerves, oral tissues, skin, and other organs.

## Data Availability

The original contributions presented in the study are included in the article/Supplementary Material, further inquiries can be directed to the corresponding author.
