# Supplementary material for: Injectable hybrid nanofibrous spheres made of PLA and nano-hydroxyapatite for cell delivery and osteogenic induction
Source: Front Bioeng Biotechnol. 2024 Aug 29;12:1460870. doi: 10.3389/fbioe.2024.1460870 (PMC11394597; doi:10.3389/fbioe.2024.1460870)
Supplement: Supplementary file 1 [file DataSheet1.docx]

**Supplementary Material**

**Injectable hybrid nanofibrous spheres made of PLA and nano-hydroxyapatite for cell delivery and osteogenic induction**

Yawen Wang^1,2,3^, Xiaopei Zhang^1,2,3^, Na Liu^2,3^, Renjie Chen^4^, Chenghao Yu^2^, Lijie Yao^1,2,3^, Siyu Chen^1,2,3^, Yuying Yan^1,2,3^, Tong Wu^1,2^, Yuanfei Wang^5^*

^1^ Shandong Key Laboratory of Medical and Health Textile Materials, Collaborative Innovation Center for Eco-textiles of Shandong Province and the Ministry of Education, College of Textile & Clothing, Qingdao University, Qingdao 266071, China.

^2^ Medical Research Center, The Affiliated Hospital of Qingdao University, Qingdao University, Qingdao 266000, China.

^3^ Institute of Neuroregeneration & Neurorehabilitation, Department of Pathophysiology, School of Basic Medicine, Qingdao University, Qingdao, 266071, China.

^4^ Beijing Jishuitan Hospital, Capital Medical University, Beijing 100035, China.

^5^ Qingdao Stomatological Hospital Affiliated to Qingdao University, Qingdao 266001, China.

* Corresponding author: [zhizunbao19@163.com](mailto:zhizunbao19@163.com) (Yuanfei Wang)


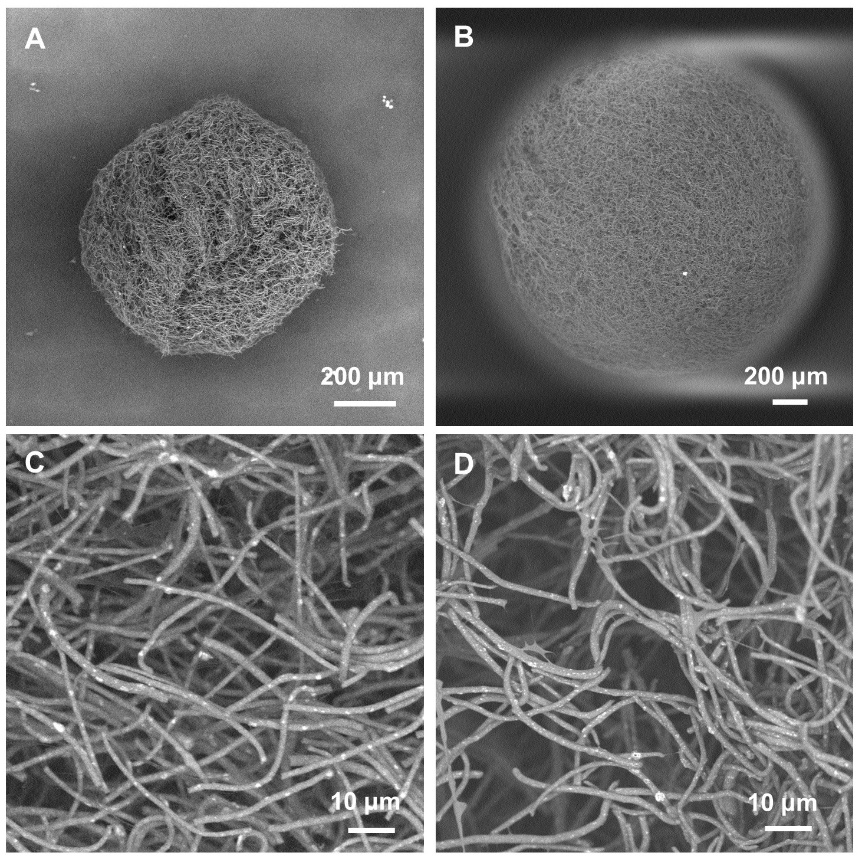


**Figure S1.** SEM images of the nanofibrous spheres prepared with PLA/nHA hybrid nanofibers of 5% nHA in different sizes: (A) the nanofibrous spheres with a diameter of approximately 750 μm; (B) the nanofibrous spheres with a diameter of approximately 1750 μm. (C, D) SEM images of the magnified view of the nanofibrous spheres in (A) and (B).
